# The Influence of Coinfection on Mood States in HTLV-1-Infected Patients

**DOI:** 10.5402/2012/325864

**Published:** 2012-07-01

**Authors:** Maria Rita Polo Gascón, Claudio Garcia Capitão, Maria Cezira Fantini Nogueira-Martins, Jorge Casseb, Augusto Cesar Penalva Oliveira

**Affiliations:** ^1^Psychology Division, Hospital das Clínicas of the Medical College of São Paulo and ICHC/FMUSP, 05403-000 São Paulo, SP, Brazil; ^2^Instituto de Infectologia Emílio Ribas and University of São Francisco, Itatiba, São Paulo, Brazil; ^3^Health Institute of São Paulo, São Paulo, Brazil; ^4^Laboratory of Dermatology and Immunodeficiencies (LIM-56), Medical School of the University of São Paulo, São Paulo, Brazil; ^5^Instituto de Infectologia Emílio Ribas, São Paulo, Brazil

## Abstract

The objective of this study was to discuss the influence of coinfection on mood states (depression and anxiety) in Human T Lymphotropic virus type 1 HTLV-1-infected patients. A cross-sectional study was performed with a sample obtained through a nonprobabilistic technique. A total of 130 patients in treatment at the HTLV Ambulatory of Instituto de Infectologia Emílio Ribas participated in the research, of whom 63 had HAM/TS and 67 were asymptomatic. A sociodemographic survey and the Beck Anxiety and Depression Inventories were used. The results indicated a prevalence of 7.2% for HTLV-1/HIV co-infection, 7.2% for HTLV-1/HCV, and 4.0% for HTLV-1/HIV/HCV. It is possible that the presence of a co-infection causes greater fear and concern about the future than asymptomatic HTLV-1 infection, increasing the observed degree of depression and anxiety.

## 1. Introduction

The HTLV virus is a retrovirus that was first isolated in 1980 from a patient with a rare type of T-cell leukemia. The disease has two subtypes: HTLV-1, which is associated with neurological disease (tropical spastic paraparesis, or HAM/TSP) and adult T-cell leukemia (ATL), and HTLV-2, which has not been shown to cause disease [1]. Other pathologies have also been linked to HTLV-1, including poliomyositis, polyarthritis, uveitis, and infective dermatitis in children. ATL was first described in Japan by Uchiyama et al. in 1977 [2] and has since been reported in many other parts of the world. HTLV-1 was isolated for the first time in T cells derived from lymph nodes and lymphocytes from the peripheral blood of a patient with cutaneous lymphoma. The connection between the virus and T-cell leukemia was established in 1982, and soon after, many reports demonstrated that the virus is also associated with other human diseases, the most notable being HAM/TSP [3].

HTLV-1 infection is endemic in southwest Japan, the Caribbean Basin, Melanesia, and parts of Africa and Brazil. In some areas, the prevalence rate is as high as 15% of the general population. In the USA, the average rate of seroprevalence of HTLV-1 and HTLV-2 among volunteer blood donors is approximately 0.016% [1, 4]. It is estimated that 15 to 20 million people carry HTLV-1 worldwide [5]. In Brazil, according to data from the Ministry of Health [6], 750,000 people are infected with HTLV.

The impact of HTLV-1 on patients' mood states has received attention from researchers; the effects have been examined in terms of the severity and chronicity of the symptoms and the prevalence of infections at the national and international levels. Preliminary results from an open cohort study of the prevalence of seropositive blood donors at a hemocenter in Minas Gerais, Brazil, suggested a higher rate of depression in HTLV-1-infected individuals compared to seronegative blood donors (45.5% versus 18.8%; *P* = 0.0543) [7].

In a study by Souza [8] of 36 HTLV-1-infected patients, depressive symptoms were observed in 10 of the patients (28%); 20% had tropical spastic paraparesis and 7.7% were asymptomatic. The author stresses that 77% of the interviewed patients described at least one symptom of depression, most often sleep disorders, lack of appetite, and anhedonia.

Another study conducted in Salvador, Brazil, by de Carvalho et al. [9], examined 50 HTLV-1-infected patients, 26 of whom were symptomatic and 24 of whom were asymptomatic. Among the evaluated patients, 21 presented with psychiatric comorbidities (42%), 17 presented with mood alterations (34%), and 11 presented with anxiety (22%). 

Gascón [10] studied 130 HTLV-infected patients in São Paulo, Brazil; 67 were asymptomatic, and 63 had HAM/TSP. Symptoms of depression were observed in 59.3% of the patients with HAM/TSP and 20.8% of the asymptomatic patients. The prevalence of anxiety was 55.5% in patients with HAM/TSP and 25.3% in asymptomatic patients. According to the authors, the main factors influencing the results were related to social characteristics, including education, family income and social class, social and familial support, clinical aspects, and the duration of infection. Another interesting factor affecting the severity of depression and anxiety was the existence of a coinfection and its associated symptoms, mostly among the asymptomatic patients.

HTLV can be transmitted sexually, parenterally, or vertically; the most effective route, when compared to HIV (Human Immunodeficiency Virus) and HVC (Hepatitis C Virus) transmission, is through breastfeeding [11]. Both HIV and HVC are easily transmitted, causing frequent coinfections. HVC coinfection is a public health problem due to its high frequency, changes in the clinical and laboratorial courses of both infections, and psychosocial repercussions. According to Rêgo et al. [12], a viral infection predisposes the carrier to the acquisition of other diseases.

Coinfection with other viruses, such as HIV and HVC, is a subject of public health interest due to effects on physical and emotional health. The data suggest that HIV patients who are co-infected with HTLV-1 have higher probabilities of developing neurological disease than do the monoinfected patients [11]. Other studies suggest that the presence of HTLV-1 infection can raise the risk of carcinoma due to the acceleration of liver disease by HVC. Co-infected patients are 2.4 times more likely to develop diseases related to HVC than mono-infected patients are [13].

One of the first reports of HIV/HTLV coinfection raised the possibility of faster disease progression among homosexual males in Trinidad and Tobago [14]. In a study conducted in Bahia, Brazil, by Brites et al. [15], a higher rate of AIDS was observed in co-infected women compared to those infected only by HIV-1. A retrospective analysis by the same author compared 63 co-infected patients with 126 patients infected only by HIV-1, revealing a shorter survival time for the co-infected group (1849 average days compared to 2430 average days for the mono-infected patients). The difference remained significant after adjusting for the use of intravenous drugs.

Coinfection thus contributes to physical damage and intense psychological suffering due to the increased possibility of death and the threat to the integrity of the ego [16, 17]. The purpose of this paper is to discuss the influence of the presence of a coinfection on the mood states (depression and anxiety) of patients infected by the HTLV-1 virus.

## 2. Material and Methods

A cross-sectional design was used for this study. The participant pool was composed of volunteers, and sampling was conducted using a non-probabilistic technique (intentional sampling by convenience). The study was conducted at Instituto de Infectologia Emílio Ribas in São Paulo, Brazil, from May 2008 to July 2009. The sample group consisted of 130 adults with HTLV-1; 67 were asymptomatic, and 63 were co-infected with HAM/TSP.

All of the patients were in clinical treatment for HTLV at Instituto de Infectologia Emília Ribas and were invited to participate in the research. A contact was established within the medical team responsible for the HTLV Clinic to identify patients fitting the research profile. According to the ethical precepts governing research with human subjects, the participants provided informed consent once they had been informed of the objectives of the research.

A sociodemographic survey, the Beck Depression Inventory, and the Beck Anxiety Inventory were used to measure mood states in the participants. It is important to note that although the depression and anxiety inventories are self-report forms, the measures were read aloud by the researcher due to the low educational levels of most of the participants. The administration took, on average, 45 minutes. The sociodemographic questionnaire was created by the researchers. The questionnaire contained 17 items related to age, gender, relationship status, educational level, and family income.

The Beck Depression Inventory (BDI; [18]) is probably the most frequently used self-report evaluation of depression in both research and clinical settings [19]; it has been translated into several languages and validated in different countries. The original scale consists of 21 items measuring symptoms and attitudes, with intensity varying from 0 to 3. The items refer to sadness, pessimism, sense of failure, lack of satisfaction, sense of guilt, sense of punishment, self-hatred, self-accusation, suicidal ideation, crying spells, irritability, social retraction, indecision, distortion of body image, inhibition of work, sleep disorder, fatigue, loss of appetite, weight loss, somatic worry, and libido reduction.

For the evaluation of anxiety, the Beck Anxiety Inventory (BAI; [20]) was used to assess characteristic symptoms of anxiety. The inventory is composed of 21 items related to the presence of anxiety symptoms [21]. For each item, subjects rate the intensity of the anxiety symptom from 0 to 3. The lowest rating, 0, corresponds to “absent”; 1 corresponds to “slight, does not bother me much”; 2 corresponds to “moderate, it is unpleasant, but I can stand it”; 3 corresponds to “severe, I almost cannot stand it.” If a subject selected more than one rating, the more intense score was always registered.

After processing, the results were organized in tables and cross-tabulated when appropriate. The raw values and percentages were used in the analysis.

The data were analyzed using descriptive statistics, including percentages, averages, frequencies, and the *rho *of Spearmann. The variables considered were the presence of coinfection and the results of the Beck Depression and Anxiety Inventories, with a type-1 error probability (alpha) of 5%. This study observed the rules of Resolution no. 196 of October 10th, 1996, of Conselho Nacional de Saúde (National Health Council), approved by Comitê de Ética em Pesquisa do Instituto de Infectologia Emílio Ribas (Ethics Committee on Research of Emilio Ribas Institute of Infectology) in October 2007.

## 3. Results

In total, 93 patients in the study sample were females (71.5%) and 37 were males (28.4%). The participants had a mean age of 49.8 years (SD = 11.6 years). Most of the participants were whites (39.2%), married (32.3%), had completed 0–4 years of education (34.6%), and hand manual occupations (30%). On Osame's scale, 21 patients had partial incapacity to walkwithout assistance, 31 needed hand support to walk, and 11 patients had complete walking incapacity.

The prevalences of various coinfections in the sample were *n* = 9 (7.2%) for HTLV-1/HVC; *n* = 8 (6.4%) for HTLV-1/HIV; *n* = 6 (4.8%) for HTLV-1/HIV/HVC; *n* = 1 (0.8%) for HTLV-1/HBV; *n* = 1 (0.8%) for HTLV-1/HIV/HBV. Among the patients who were co-infected with HCV, eight presented with HAM/TSP, and one was asymptomatic; among those co-infected with HIV, most were asymptomatic carriers of HTLV-1 (*n* = 7), and only one presented with HAM/TSP. Among those co-infected with HTLV-1/HVC/HIV, four presented with HAM/TSP, and two were asymptomatic.

Most of those interviewed (*n* = 77; 59.2%) were unaware of their mode of infection. Based on the common sources of infection, we determined that sexual intercourse (*n* = 22, 16.9%), blood transfusions (*n* = 17, 13.1%), breastfeeding (*n* = 10, 7.7%), use of endovenous drugs (*n* = 3, 2.3%), and tattoos (*n* = 1, 0.8%) were the main modes of infection. In the group of co-infected patients, 50% (*n* = 12) were unaware of their most likely source of infection; 33.3% reported sexual intercourse as the source (*n* = 8), while 8.3% named blood transfusions and use of injectable illicit drugs (*n* = 2).

The frequencies of moderate and severe depression were 40.3% and 19.0%, respectively (59.3% overall), in the HAM/TSP group and 13.5% and 8.9%, respectively (32.3% overall), in the control group (Table 1). The most prevalent symptoms were fatigue (74.6%), health concerns (73.8%), irritability (65.3%), sleep disturbances (63.8%), difficulty working (61.5%), reduction in or lack of sexual desire (57.6%), apathy (54.7%), self-criticism (53.8%), and anhedonia (53%).

The combined frequency of moderate (31.7%) and severe (23.8%) anxiety was 55.5% in the HAM/TSP group and 14.9% (moderate: 10.4%, severe: 25.3%) in the control group (Table 2). The most prevalent symptoms were nervousness (63.8%), numbness or tingling (59.2%), difficulty relaxing (56.2%), lack of balance (55.4%), and fear of losing control (52.3%).

The analysis revealed impacts on mood state caused by the presence of a coinfection within the group of asymptomatic patients; both social and psychological vulnerabilities were observed to a greater extent in this group than among the mono-infected patients. These results are shown in Table 3.

## 4. Discussion

The analysis demonstrated that the presence of a coinfection had significant effects on the severity of depression and anxiety in the asymptomatic group. Infection by HIV or HVC may make the individual more vulnerable, encouraging him or her to perseverate on topics such as death. In patients who show symptoms of the diseases caused by these viruses, the illusions of self-sufficiency and immortality are confronted by a real threat. It is important to understand these psychological effects because HIV and the liver diseases caused by HVC are worldwide epidemics that have already killed many people.

The infections and threat of death caused by these viruses can increase patients' feelings of devaluation, fear, regret, and guilt, among other emotions; this experience causes the coinfection to be perceived as a threat that the individual cannot escape. Co-infected patients experience additional suffering due to social discrimination, multiple losses (e.g., of a healthy body, relationships, occupation, leisure activities), and often the need to live a double life to avoid discrimination [17].

In addition to the distress caused by the multiple losses that a patient incurs following the diagnosis of a coinfection, another type of distress is present in this context: the distress of castration. The real possibility of sickness and death is strongly linked to castration as it subtracts from the possibility of life and the fantasy of immortality. The distress of castration comes from the fear of separation from something extremely valuable, such as family and friends; therefore, the distress of castration can be defined as a reaction to situations involving danger and threat to the integrity of the subject. We can conclude that the co-infected patient's self remains absolutely vulnerable and helpless when facing possible death [16].

The results within the group of asymptomatic patients can be translated as fear and apprehension among both patients that the severity of symptoms may worsen in the future.

## 5. Conclusion

The results of the present study suggest that the presence of a coinfection in asymptomatic HTLV-1 patients is related to feelings of fear and apprehension about the future, in addition to depressive symptoms.

The experimental portion of the research was exploratory and cross-sectional, allowing us to examine a rarely investigated theme: factors related to coinfections in the carriers of HTLV-1. We can conjecture that other factors influence the lives of these patients. This study can contribute to future research on the factors that were not studied, such as the psychosocial and organic factors related to coinfection and depression. Together with the findings of the present work, future research can guide the development of supportive interventions and protocols to promote the physical and mental health of patients infected with HTLV-1.

In conclusion, the evidence provides important information for health professionals about the realities and vulnerabilities of HTLV-1 patients and about the possibility of their exposure to other viruses. Furthermore, it is the intention of this study to arouse the interest of authorities, professionals, and health researchers in systemic, multidisciplinary interventions providing clinical, epidemiological, psychological, and social support.

## Figures and Tables

**Table 1:** Frequency of depression in 130 patients treated at the Emílio Ribas Infectious Diseases Institute from May 2008 to July 2009.

Level of depression (BDI)	All patients (*n* = 130)	Symptomatic (*n* = 63)	Asymptomatic (*n* = 67)
Minimal	59 (45.4%)	18 (28.5%)	41 (61.1%)
Slight	19 (14.6%)	8 (12.6%)	11 (16.4%)
Moderate	34 (26.2%)	25 (40.3%)	9 (13.6%)
Severe	18 (13.8%)	12 (19%)	6 (8.9%)

**Table 2:** Frequency of anxiety in 130 patients treated at the Emílio Ribas Infectious Diseases Institute from May 2008 to July 2009.

Level of anxiety (BAI)	All patients (*n* = 130)	Symptomatic (*n* = 63)	Asymptomatic (*n* = 67)
Minimal	58 (44.6%)	15 (23.8%)	43 (64.1%)
Slight	20 (15.4%)	13 (20.6%)	7 (10.4%)
Moderate	30 (23.1%)	20 (31.7%)	10 (14.9%)
Severe	22 (17%)	15 (23.8%)	7 (10.4%)

**Table 3:** Spearman's correlation coefficient and *P* values for asymptomatic co-infected patients.

	Spearman's *ρ*	*P* value
Depression	0.030	0.266
Anxiety	0.046	0.245

Level of significance: *P* < 0.05.

## References

[1] Casseb J, Oliveira ACP (2008). Low risk for TSP/HAM development after 50 years old. *Clinical Infectious Diseases*.

[2] Uchiyama T, Yodoi J, Sagawa K (1977). Adult T-cell leukemia: clinical and hematologic features of 16 cases. *Blood*.

[3] Soares BCC, Proeietti FA, Proietti ABFC (2001). Os vírus linfotrópicos de células T Humanos (HTLV) na última década (1990–2000): Aspectos Epidemiológicos. *Revista Brasileira de Epidemiologia*.

[4] Carneiro-Proetti ABF, Ribas JGR, Catalan-Soares BC (2002). Infecção e doença pelos vírus linfotrópicos humanos de células T (HTLV-I/II) no Brasil. *Revista da Sociedade Brasileira de Medicina Tropical*.

[5] Edlich RF, Arnette JA, Williams FM (2000). Global epidemic of human T-cell lymphotropic virus type-I (HTLV-I). *Journal of Emergency Medicine*.

[6] Brasil

[7] Stumpf BP, Catalan-Soares B, Carneiro-Proietti AB, Namen-Lopes S, Proietti FA, Rocha FL (2005). Depression in HTLV-1 infected individuals: initial reports from the GIPH cohort in Belo Horizonte, Brazil. *AIDS Research and Human Retroviruses*.

[8] Souza ARM (2009). Frequency of major depressive disorder in HTLV-I infected patients. *Arquivos de Neuro-Psiquiatria*.

[9] de Carvalho AGJ, Galvão-Phileto AV, Lima NS, de Jesus RS, Galvão-Castro B, Lima MG (2009). Frequency of mental disturbances in HTLV-1 patients in the State of Bahia, Brazil. *Brazilian Journal of Infectious Diseases*.

[10] Gascón MRP, Capitão CG, Casseb J, Nogueira-Martins MCF, Smid J, de Oliveira ACP (2011). Prevalence of anxiety, depression and quality of life HTLV-1 infected patients. *Brazilian Journal of Infectuos Diseases*.

[11] Brites C, Cosmo C, Oliveira A (2007). Co-Infecção HIV-HTLV. *Tendências em HIV-AIDS*.

[12] Rêgo A, Feitosa F, Cavalcante D, Paraná D (2003). VHC e HTLV-I: aspectos clínicos e epidemiológicos da co-infecção. *Revista de Ciências Médicas e Biológicas*.

[13] Bermúdez-Aza E, Kallás EG (2007). Epidemiologia da Co-Infecção HIV e HCV. *Tendências em HIV-AIDS*.

[14] Bartholomew CD, Blattner W, Cleghorn F (1987). Progression to aids in homosexual men co-infected with HIV and HTLV-I in Trinidad. *The Lancet*.

[15] Brites C, Harrington W, Pedroso C, Netto E, Badaró R (1994). HIV 1 and HTLVI/II co-infection in Brazil: IV drug use the major route of transmission. *AIDS Research and Human Retroviruses*.

[16] Freud S (1920). Além do Princípio de Prazer. *Standard Brasileira*.

[17] Kubler-Ross E (1989). *Sobre a Morte e Morrer*.

[18] Beck AT, Ward CH, Mendelson M, Mock J, Erbaugh J (1961). An inventory for measuring depression. *Archives of General Psychiatry*.

[19] Dunn G, Sham P, Hand D (1993). Statistics and the nature of depression. *Psychological Medicine*.

[20] Beck AT, Epstein N, Brown G, Steer RA (1988). An inventory for measuring clinical anxiety: psychometric properties. *Journal of Consulting and Clinical Psychology*.

[21] Cunha JÁ (2001). *Manual da versão em português das escalas de Beck. Tradução e Adaptação Brasileira*.

